# Epidermal growth factor strongly affects epithelial Na^+^ transport and barrier function in fetal alveolar cells, with minor sex-specific effects

**DOI:** 10.1038/s41598-021-95410-y

**Published:** 2021-08-05

**Authors:** Mandy Laube, Diana Dornis, Fine Wenzel, Ulrich H. Thome

**Affiliations:** Department of Pediatrics, Division of Neonatology, Center for Pediatric Research Leipzig (CPL), University of Leipzig, Liebigstrasse 19, 04103 Leipzig, Germany

**Keywords:** Respiration, Permeation and transport, Paediatric research, Intrauterine growth

## Abstract

Male sex remains an independent risk factor for respiratory distress syndrome (RDS) in preterm infants. Insufficient Na^+^ transport-mediated alveolar fluid clearance contributes to RDS development and we previously demonstrated sex-specific differences in Na^+^ transport. The epidermal growth factor (EGF) is important during fetal lung development with possible influence on Na^+^ transport. Sex-specific effects of EGF during surfactant synthesis were shown. We thus determined whether EGF exerts sex-specific effects on Na^+^ transport in fetal alveolar cells. We analyzed sex-specific fetal distal lung epithelial (FDLE) cells exposed to EGF and related ligands with Ussing chambers, RT-qPCR and Western blots. EGF strongly reduced the epithelial Na^+^ channel (ENaC) mRNA levels in both male and female FDLE cells. This was corroborated by a markedly reduced ENaC activity, while amiloride-insensitive pathways as well as barrier function were raised by EGF. In contrast to chronic effects, acute effects of EGF were sex-specific, because Na^+^ transport was reduced only in males. AKT phosphorylation was elevated only in female cells, while pERK1/2 was increased in both male and female cells. EGF showed certain sex- and time-dependent effects in FDLE cells. Nevertheless, the results suggest that EGF is an unlikely cause for the sex-specific differences in Na^+^ transport.

## Introduction

During fetal development, lung epithelial cells actively secrete fluid, thereby filling the developing lung. Vectorial Cl^−^ transport-driven pulmonary fluid accumulation supports lung growth by establishing an intra-pulmonary pressure that promotes cellular proliferation. Animal studies with intrauterine tracheal drainage as well as tracheal occlusion demonstrated the pivotal relationship between fluid accumulation and fetal lung development^1^. A contribution of the cystic fibrosis (CF) transmembrane conductance regulator (CFTR) to this process has been suggested^2–4^. More precisely, during fetal lung development CFTR expression exhibits a time- and tissue-dependent expression pattern. The highest CFTR expression level was observed in the 1st and 2nd gestational trimester, while its expression gradually declines during the 3rd trimester^5,6^. Epithelial cell proliferation was accelerated by *Cftr* over-expression in the pseudoglandular stage resulting in an enhanced lung growth^3^. Prior to birth, the fetal lung fluid has to be removed to enable air breathing. Alveolar fluid clearance (AFC) is driven by epithelial Na^+^ transport accomplished by epithelial Na^+^ channels (ENaC) in the apical membrane compartment and the Na,K-ATPase in the basolateral membrane compartment of alveolar type II (ATII) cells. ENaC consist of three homologous subunits, α-, β-, and γ-ENaC^7^, and the Na–K-ATPase is composed of α_1_- and β_1_-subunits in ATII cells^8^. Vectorial Na^+^ transport establishes an osmotic driving force causing fluid absorption from the air spaces into the interstitium. In premature newborns a decreased AFC has been shown^9^, possibly due to a lower expression of epithelial Na^+^ channels^10^. In addition to surfactant deficiency, AFC insufficiency contributes to the development of the respiratory distress syndrome (RDS)^10^. Importantly, a sex ratio of 1:1.7^11,12^ was observed for the RDS incidence, with males developing RDS significantly more frequently compared with female infants of the same gestational age, raising male mortality^13^. Up until now, male sex remains an independent risk factor for RDS development^11,12^. We have previously shown sex-specific differences in alveolar Na^+^ transport. Male sex was associated with lower Na^+^ transport and reduced levels of the ENaC and Na–K–ATPase subunits in fetal distal lung epithelial (FDLE) cells^14^. Na^+^ transport in female cells was more responsive to female sex steroids possibly contributing to the observed female advantage^15^, while androgens and glucocorticoids were either lacking any effect or equally stimulated Na^+^ transport in both sexes^15,16^. Another important growth factor during fetal lung development is the epidermal growth factor (EGF). EGF, via its receptor (EGFR), enhances terminal branch formation and stimulates proliferation and differentiation of epithelial and mesenchymal cells in culture^17^. EGFR deficient mice exhibit a neonatal lethal pulmonary phenotype and present a reduced branching as well as condensed lungs with collapsed alveoli, resembling a human neonatal RDS phenotype^18,19^. EGFR is the prototypic member of the ErbB (erythroblastic oncogene B) family of receptor tyrosine kinases (reviewed in^20^). To date, three other members of this family have been identified: ErbB2, ErbB3, and ErbB4, which are capable of forming homodimers, heterodimers, and possibly higher-order oligomers. Despite their large degree of structural homology, ErbB family members differ from each other in their patterns of expression, ligand specificity, and intracellular substrates. Complementing these receptors is a family of ligands: e.g. EGF and transforming growth factor-α (TGF-α) bind only to ErbB1 (EGFR), heparin-binding EGF (HB-EGF) binds to both ErbB1 and ErbB4, and neuregulin 1 (NRG1) which binds to ErbB3 and ErbB4. ErbB2 has no known ligand and acts through dimerization with one of the other three receptors, while ErbB3 is kinase impaired. Downstream ErbB signaling includes phosphoinositide 3-kinases (PI3-K)/ protein kinase B (AKT), mitogen-activated protein kinase (MAPK) signaling, and the phospholipase C (PLC) pathway^21^, all of which are known to affect ENaC functions.

Sex-specific responses to EGF have been previously noted regarding surfactant synthesis^22–24^ and an impact on epithelial Na^+^ transport has been described by others^25,26^. However, a potential sex-specific effect of EGF on Na^+^ transport in fetal alveolar cells is currently unknown. We therefore aimed to determine the impact of EGF and its related ligands on Na^+^ transport in sex-specific FDLE cells and reveal contributing pathways in this context.

## Methods

### Cell isolation and culture

Sprague–Dawley rats were obtained from the Medical Experimental Center (MEZ) of Leipzig University. Animals were kept in rooms with a 12 h light–dark cycle, constant temperature (22 °C) and humidity (55%). Food and water were supplied ad libitum*.* At gestational day E20-21 (term E = 22) pregnant rats were anesthetized by CO_2_ inhalation and euthanized by Pentobarbital injection. Separation of male and female fetuses was done through visual determination, as described before^14^. All experimental procedures were approved by the institutional review board (IRB: Landesdirektion Leipzig, permit number: T23/15) and complied with the ARRIVE guidelines. All methods were carried out in accordance with relevant guidelines and regulations.

FDLE cells, a model of fetal ATII cells, were isolated from fetal lungs as described previously^27,28^. Male and female fetuses were derived from the same litter in equal numbers and prepared separately. Fetal lungs were mechanically dissociated with razor blades. The resulting cell suspension was enzymatically digested by incubation with 0.125% trypsin (Fisher Scientific, Schwerte, Germany) and 0.4 mg/ml DNAse (CellSystems, Troisdorf, Germany) in HBSS (Fisher Scientific) for 10 min at 37 °C, followed by MEM containing 0.1% collagenase (CellSystems) and DNAse for 15 min at 37 °C. To separate adjacent lung fibroblasts from FDLE cells, the cell mix was plated twice for one hour at 37 °C in cell culture flasks. FDLE cells were then seeded on permeable Snapwell inserts (Costar, # 3407, surface area 1.1 cm^2^, Corning, NY) at a density of 10^6^ cells per insert. For RNA and protein isolation, cells were seeded on larger inserts (ThinCert, #657641, surface area 4.6 cm^2^, Greiner Bio-One, Frickenhausen, Germany) at a density of 2 × 10^6^ cells per insert. To generate sex-specific epithelial-fibroblast co-cultures, FDLE cells were seeded on inserts as described above, while the adjacent lung fibroblasts obtained by differential adhesion were seeded in the compartment below the insert at the bottom of the well plate. FDLE cells were cultured in serum-supplemented medium consisting of MEM with 10% FBS (Biochrom, Berlin, Germany), glutamine (2 mM, Fisher Scientific) and antibiotic–antimycotic (Fisher Scientific). Cell culture medium was changed to serum-free medium (SF-Med, Cellgro, Mediatech, Herndon, VA, USA) supplemented with either EGF (10 ng/ml, # 354,052, BD-Bioscience, San Jose, CA, USA), HB-EGF (50 ng/ml, # 97560, Biomol, Hamburg, Germany), TGF-α (10 ng/ml, # T8250-05, Biomol) or NRG1 (50 µg/ml, # SRP3055, Sigma-Aldrich, Taufkirchen, Germany) 24 h prior to analyses. The concentrations of estradiol (E2, 0.0037 µM) and progesterone (P, 2.8 µM) were chosen based on previous studies^15,29^. The antagonist Erlotinib (10 µM, # Cay10483-250, Biomol) was used to block EGFR and thereby the EGF-evoked response. Growth factors and the receptor antagonist were dissolved in dimethyl sulfoxide (DMSO). To exclude solvent influences on the evoked responses, control cells were treated with the same amount of the respective solvent.

### Using chamber measurements

Ussing chamber measurements were performed five days after cell isolation, as previously reported^14^. Only monolayers with a transepithelial resistance (*R*_te_) exceeding 300 Ω cm^2^ were included in the analyses. Electrophysiological solutions consisted of: 145 mM Na^+^, 5 mM K^+^, 1.2 mM Ca^2+^, 1.2 mM Mg^2+^, 125 mM Cl^−^, 25 mM HCO_3_^−^, 3.3 mM H_2_PO_4_^−^ and 0.8 mM HPO_4_^2−^ (pH 7.4). For the basolateral solution, 10 mM glucose was used, while 10 mM mannitol was used in the apical solution. During measurements, the solutions were continuously bubbled with carbogen (5% CO_2_ and 95% O_2_). Short-circuit currents (*I*_SC_) were measured every 20 s with a transepithelial voltage clamp (Physiologic instruments, San Diego, CA, USA). After the *I*_SC_ reached a stable plateau (*I*_base_), amiloride (10 µM, # A7410, Sigma-Aldrich) was applied to the apical chamber to assess the amiloride-insensitive *I*_SC_ (*I*_amil-insens_). The current reduction induced by amiloride (Δ*I*_amil_) was used as a measure of ENaC activity. Forskolin (10 µM, # F-6886, Sigma-Aldrich) was added to the apical compartment after amiloride application to increase the intracellular cyclic adenosine monophosphate (cAMP) concentration and thereby activate cAMP-sensitive ion channels like CFTR, contributing to the forskolin-induced *I*_SC_ (Δ*I*_forsk_). Finally, CFTR_inh_172 (10 µM, # 3430, Bio-Techne, Wiesbaden-Nordenstadt, Germany) was applied apically to determine the CFTR_inh_172-sensitive *I*_SC_ (Δ*I*_CFTR_), a measure of CFTR activity. Amiloride was dissolved in water; forskolin and CFTR_inh_172 were prepared in DMSO.

### mRNA expression analyses

RNA isolation, reverse transcription and real-time quantitative PCR (RT-qPCR) were done as described before^16^. Gene-specific primers are listed in Table 1.

**Table 1:** Primer sequences.

Gene	Primer (forward, 5′–3′)	Primer (reverse, 3′–5′)
*α-ENaC* NM_031548.2	TTCTGGGCGGTGCTGTGGCT	GCGTCTGCTCCGTGATGCGG
*β-ENaC* NM_012648.1	TGCAGGCCCAATGCCGAGGT	GGGCTCTGTGCCCTGGCTCT
*γ-ENaC* NM_017046.1	CACGCCAGCCGTGACCCTTC	CTCGGGACACCACGATGCGG
*Na,K-ATPases-α*_*1*_ NM_012504.1	GGACGAGACAAGTATGAGCCCGC	CATGGAGAAGCCACCGAACAGC
*Na,K-ATPases-β*_*1*_ NM_012505.2	GCGCAGCACTCGCTTTCCCT	GGGCCACACGGTCCTGGTACG
*CFTR* NM_031506.1	GCCTTCGCTGGTTGCACAGTAGTC	GCTTCTCCAGCACCCAGCACTAGA
*Sftpa* NM_001270645.1	CCTCTTCTTGACTGTTGTCGCTGG	GCTGAGGACTCCCATTGTTTGCAG
*Sftpb* NM_138842.1	GGAGCTAATGACCTGTGCCAAGAG	CTGGCCCTGGAAGTAGTCGATAAC
*Sftpc* NM_017342.2	GATGGAGAGCCCACCGGATTACTC	GAACGATGCCAGTGGAGCCAATAG
*EGFR* NM_031507.1	GGGATCGGCCTCTTCATGCG	GTGCCAAATGCTCCTGAACCCAG
*Rpl13a* NM_173340.2	GGGCCATCTTCTGGGCCGC	CATGCCTCGCACAGTGCGCC

### Western blot analyses

FDLE cell inserts were placed on ice, and protein lysates prepared and analyzed as described elsewhere^30^. Phosphorylation of AKT was analyzed using antibodies against phospho-AKT at Ser473 (# 9271, Cell Signaling Technology, Frankfurt am Main, Germany), and AKT (# 9272, Cell Signaling Technology, both kindly provided by A. Garten). Phosphorylation of ERK1/2 (extracellular-signal regulated kinases) was analyzed using antibodies against phospho-ERK1/2 at Tyr202/Tyr204 (# 9101, Cell Signaling Technology), and ERK1/2 (# 9102, Cell Signaling Technology, both kindly provided by A. Garten). EGFR (C74B9, # 2646, Cell Signaling Technology) expression was measured and α-Tubulin (11H10, # 2125, Cell Signaling Technology) expression was used as a reference.

### MTT

Metabolic activity in male and female FDLE cells stimulated with EGF was determined with 3-(4,5-dimethylthiazol-2-yl)-2,5-diphenyltetrazolium bromide (MTT) assays as described before^31^.

### Cell permeability assay

Epithelial permeability was assessed with FDLE cells cultured on Snapwell inserts. After two days in culture, the medium was changed to EGF-containing or control medium. After 24 h the medium was replaced by phenol red-free MEM (Fisher Scientific) in the lower compartment and phenol red-free MEM containing FITC-dextran (0.25 mg/ml, 3–5 kDa, # 46944, Sigma-Aldrich) in the upper insert compartment. Cells were incubated for another 24 h, followed by analysis of FITC-dextran fluorescence intensity in the lower compartment.

### Statistical analysis

Differences between two groups were analyzed with the unpaired *t*-test. Otherwise, significant differences were determined by ANOVA with Dunnett’s post hoc test. A probability of *p* < 0.05 was considered significant for all statistical analyses. Statistical analysis was performed with GraphPad Prism software (GraphPad Prism 9.1.1, https://www.graphpad.com, GraphPad Software, La Jolla, CA, USA).

## Results

### Effect of EGF on Na^+^ and Cl^−^ transporters in sex-specific FDLE cells

**Figure 1:**
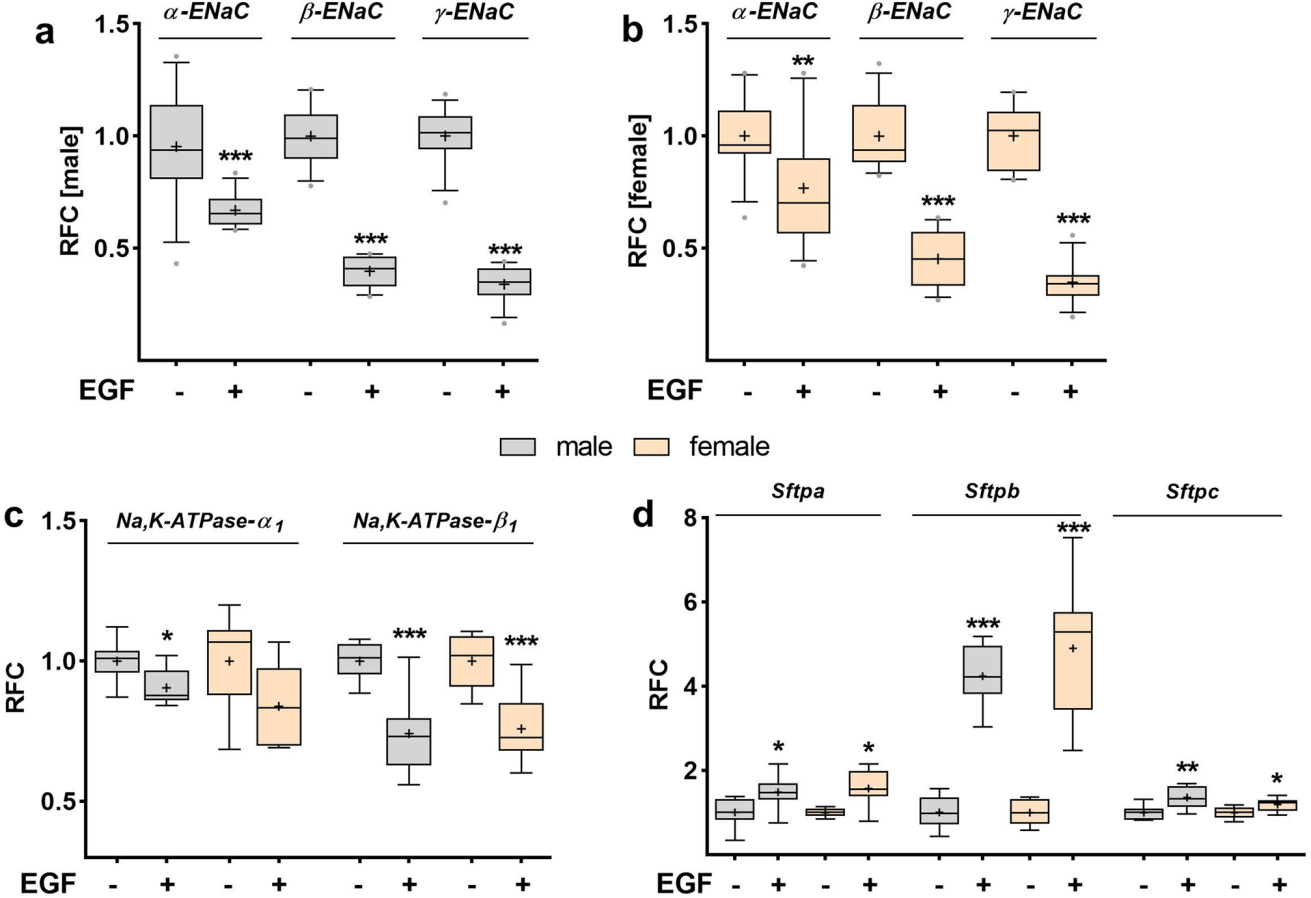
EGF reduced mRNA expression of Na^+^ transporters in both sexes. Sex-specific FDLE cells were isolated from fetal rats, cultured in SF-Med and stimulated with EGF for 24 h. Data are displayed as box and whiskers with the 10–90 percentile, mean (+) and median (horizontal line) of relative fold change (RFC). In male (**a**) and female (**b**) FDLE cells, EGF significantly decreased *α-ENaC*, *β-ENaC* and *γ-ENaC* mRNA expression (n = 11–12; **p < 0.01; ***p < 0.001, *t*-test). (**c**) EGF also reduced *Na,K-ATPase-α*_*1*_ in male FDLE cells, and *Na,K-ATPase-β*_*1*_ mRNA expression in both, male and female cells (n = 8; *p < 0.05; ***p < 0.001, *t*-test). (**d**) Surfactant protein (*Sftpa*, *Sftpb* and *Sftpc*) mRNA expression was elevated by EGF in male and female FDLE cells (n = 8; *p < 0.05; **p < 0.01; ***p < 0.001, *t*-test). (Grey boxes) male; (yellow boxes) female.

We addressed the effect of EGF on epithelial Na^+^ channels in male and female FDLE cells separately. Cells were cultured in SF-Med to exclude potential serum effects. In male and female FDLE cells, application of EGF significantly decreased *α-ENaC*, *β-ENaC* and *γ-ENaC* mRNA expression after 24 h compared with control cells (Fig. 1a, b). In detail, EGF reduced *α-ENaC* mRNA expression by approximately 30%, and *β-ENaC* and *γ-ENaC* mRNA expression by approximately 60% (*p* < 0.01; *p* < 0.001). EGF further reduced *Na,K-ATPase-α*_*1*_ in male FDLE cells by 10%, and *Na,K-ATPase-β*_*1*_ mRNA expression by 25% in both, male and female cells (*p* < 0.05; *p* < 0.001, Fig. 1c). In contrast to the reduction of Na^+^ transporter mRNA levels, EGF significantly elevated the mRNA expression of surfactant protein A (*Sftpa*), B (*Sftpb)* and C (*Sftpc*) in both sexes (Fig. 1d). Moreover, EGF increased *Sftpa* mRNA levels by 50%, *Sftpb* by more than 400%, and *Sftpc* by approximately 20–30% (*p* < 0.05; *p* < 0.01; *p* < 0.001), without any pronounced sex difference.

**Figure 2:**
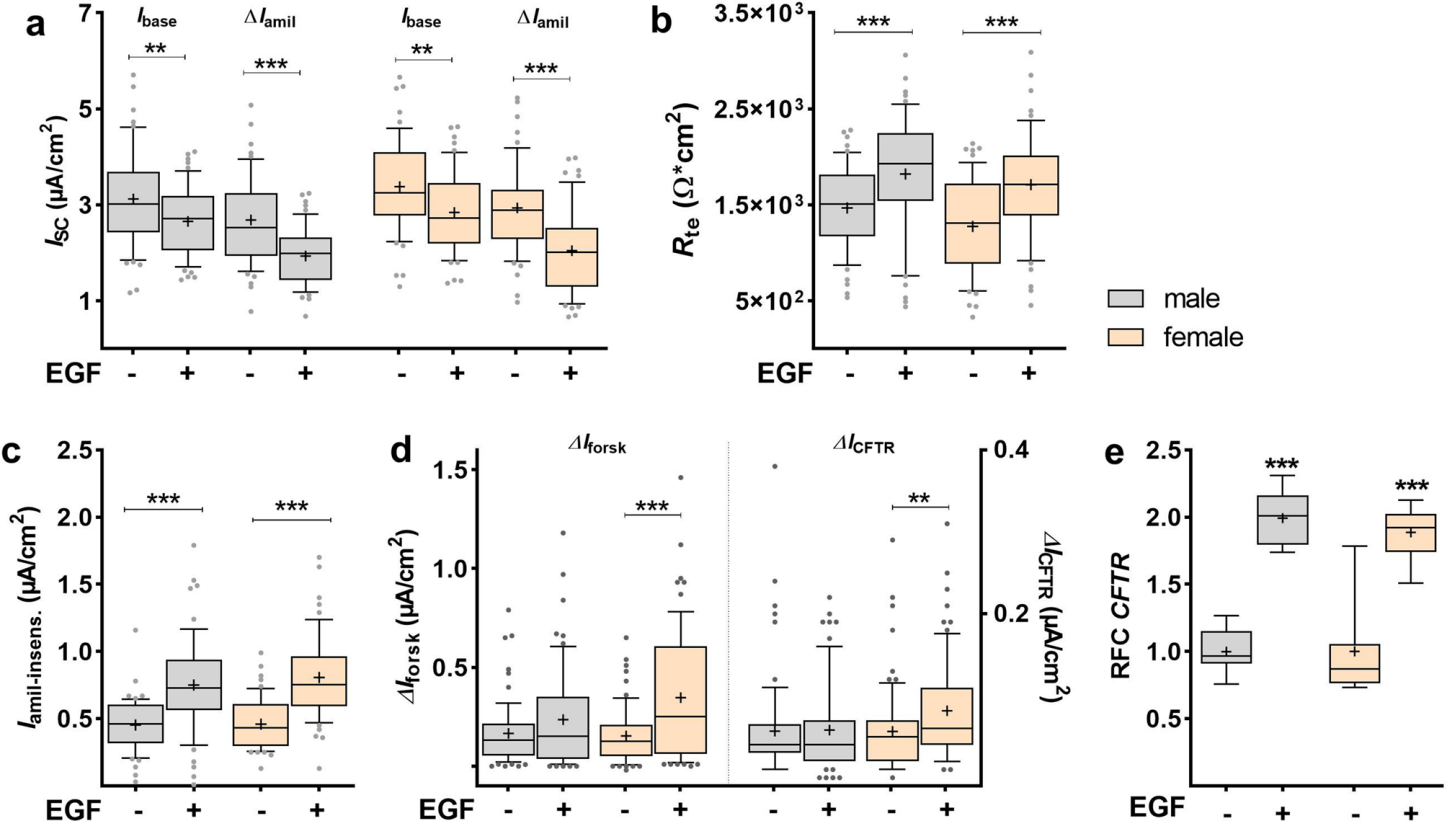
EGF decreased Na^+^ transport, but increased Cl^−^ transport and CFTR mRNA expression. FDLE cells were cultured in SF-Med and stimulated with EGF for 24 h. Data are displayed as box and whiskers with the 10–90 percentile, mean (+) and median (horizontal line). (**a**) In male and female FDLE cells, EGF significantly decreased *I*_base_ and Δ*I*_amil_ (n = 52–59; **p < 0.01; ***p < 0.001 by *t*-test). EGF further significantly increased *R*_te_ (**b**) and *I*_amil-insens_ (**c**) in both sexes (***p < 0.001). (**d**) EGF strongly increased Δ*I*_forsk_ and Δ*I*_CFTR_, but only in female FDLE cells (n = 71–76; **p < 0.01; ***p < 0.001 by *t*-test). (**e**) EGF significantly increased *CFTR* mRNA expression in both sexes (n = 8; ***p < 0.001; *t*-test). (Grey boxes) male; (yellow boxes) female.

Ussing chamber analyses demonstrated that baseline *I*_SC_ (*I*_base_) of male cells was significantly decreased by EGF after 24 h compared with untreated male controls (*p* < 0.01; Fig. 2a). The amiloride-sensitive *I*_SC_ (Δ*I*_amil_) was also significantly decreased by EGF in male FDLE cells (*p* < 0.001). Similarly, EGF significantly decreased *I*_base_ and Δ*I*_amil_ in female FDLE cells (*p* < 0.01; *p* < 0.001; Fig. 2a). The results suggest that epithelial Na^+^ transport of male and female FDLE cells is equally reduced by EGF after 24 h. In contrast, epithelial barrier function with regard to *R*_te_ was highly increased by EGF in both sexes (*p* < 0.001; Fig. 2b). Furthermore, the amiloride-insensitive *I*_SC_ (*I*_amil-insens_), most likely Cl^−^ transport, was strongly increased by EGF in male and female cells (*p* < 0.001; Fig. 2c). This is further supported by the significantly elevated forskolin-induced *I*_SC_ (Δ*I*_forsk_) and the increased CFTR_inh_172-sensitive *I*_SC_ (Δ*I*_CFTR_) induced by EGF in female FDLE cells (*p* < 0.01; *p* < 0.001; Fig. 2d). Surprisingly, male FDLE cells did not show a significant increase of CFTR activity, since Δ*I*_forsk_ and Δ*I*_CFTR_ were not affected by EGF addition. Finally, CFTR mRNA expression was strongly increased by EGF in both, male and female FDLE cells (*p* < 0.001; Fig. 2e). Taken together, EGF negatively regulated mRNA level and activity of Na^+^ transporters in both sexes after 24 h. On the other hand, EGF strongly elevated Cl^−^ transport in females and CFTR mRNA expression in both, male and female FDLE cells.

**Figure 3:**
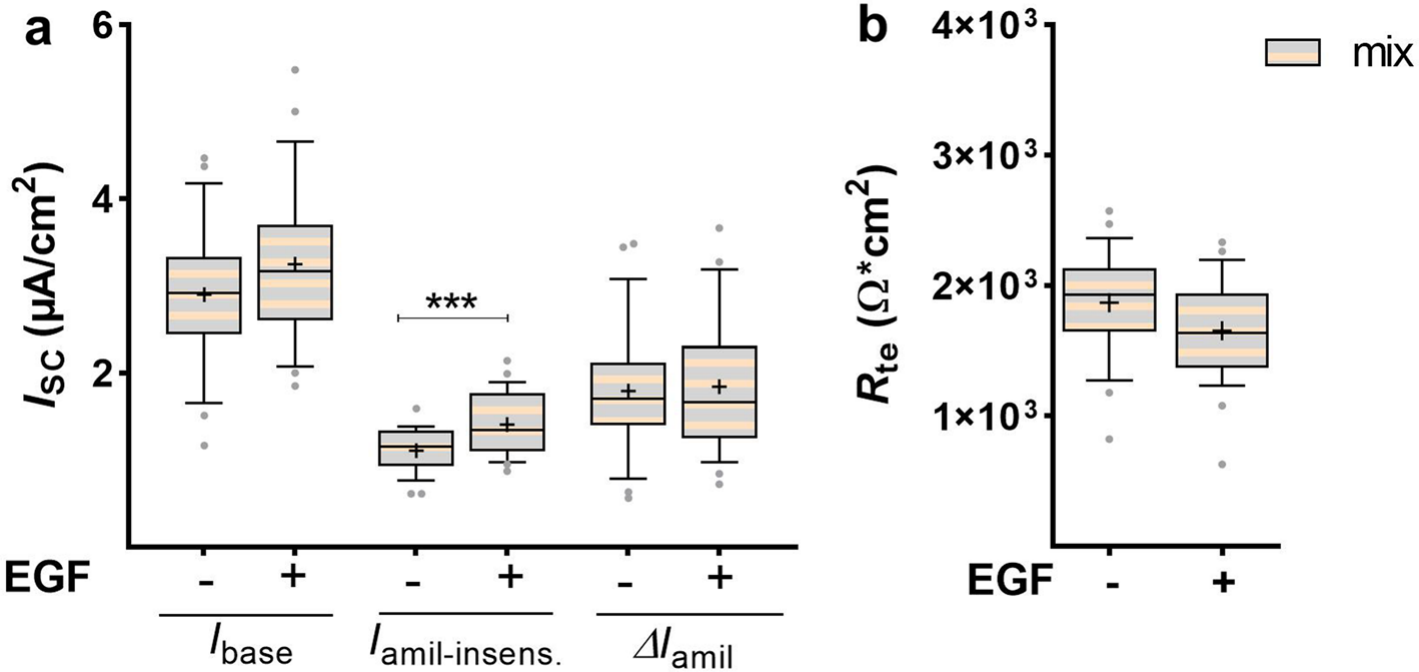
Persistence of EGFs’ effect. FDLE cells (mixed sexes) were cultured in SF-Med and stimulated with or without EGF for 24 h, followed by a medium exchange to SF-Med lacking EGF in both groups and culture for another 24 h. Data are displayed as box and whiskers with the 10–90 percentile, mean (+) and median (horizontal line). (**a**) The reducing effect of EGF on *I*_base_ and Δ*I*_amil_ did not persist after 24 h culture in SF-Med, while the increasing effect on *I*_amil-insens_ was retained in mixed FDLE cells (n = 26/27; ***p < 0.001 by *t*-test). (**b**) Furthermore, the stimulating effect of EGF on *R*_te_ was lost after 24 h in SF-Med.

Persistence of the EGF response was tested by stimulating FDLE cells with EGF for 24 h, followed by another 24 h culture in SF-Med without EGF. These experiments were done in mixed FDLE cells of both sexes. Culture with SF-Med restored *I*_base_ and Δ*I*_amil_ of initially EGF-treated cells to control levels (Fig. 3a). In contrast, EGF’s effect on *I*_amil-insens_ persisted even after 24 h culture in SF-Med, shown by the significantly higher *I*_amil-insens_ in formerly EGF-treated cells (*p* < 0.001). The elevated barrier function shown above was abolished by 24 h culture in SF-Med (Fig. 3b), suggesting that the effect of EGF on Na^+^ transport and *R*_te_ requires its continuous presence.

### Sex-specific expression of EGFR

**Figure 4:**
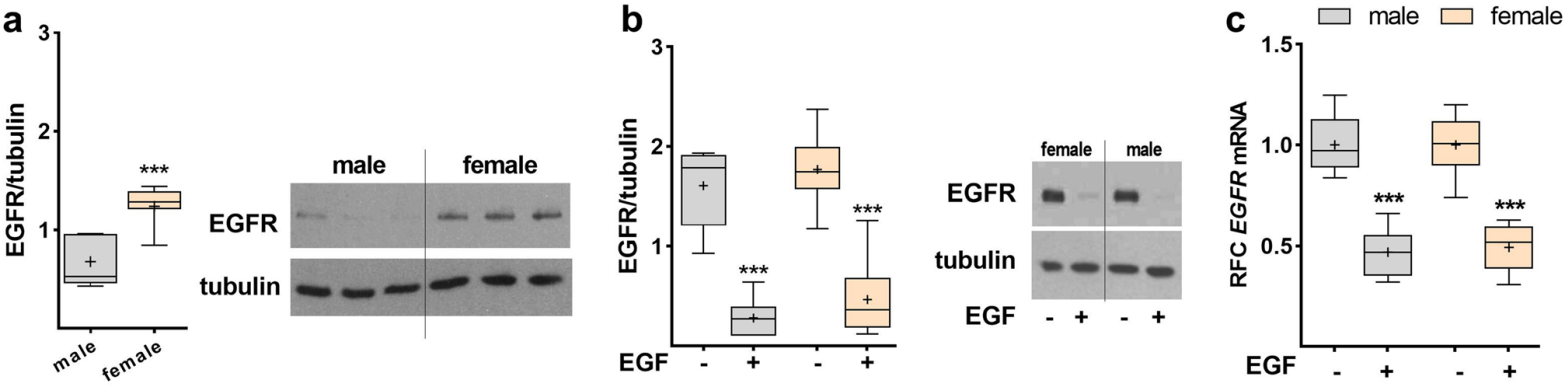
Sex-specific and EGF-dependent expression of EGFR. Data are displayed as box and whiskers with the 10–90 percentile, mean (+) and median (horizontal line). (**a**) EGFR protein expression was significantly higher in female FDLE cells compared to male cells in the presence of serum (n = 7; ***p < 0.001 by *t*-test). EGF application to FDLE cells cultured in SF-Med reduced EGFR protein (**b**) and mRNA expression (**c**) after 24 h in FDLE cells of both sexes (n = 6/8; ***p < 0.001 by *t*-test). (Grey boxes) male; (yellow boxes) female.

In the presence of fetal serum, female FDLE cells displayed a higher EGFR protein expression (*p* < 0.001; Fig. 4a). In FDLE cells cultured in SF-Med no sex-specific protein expression of EGFR was observed, and EGF application significantly downregulated EGFR protein expression after 24 h in both sexes (*p* < 0.001; Fig. 4b). In accordance, EGF also reduced EGFR mRNA expression in male and female cells by approximately 80% (*p* < 0.001; Fig. 4c), and EGFR mRNA expression was not sex-specific. The results thus suggest that EGFR expression is sex-specific only in the presence of fetal serum, while EGF equally reduces the expression of its own receptor in both sexes.

### Acute effects of EGF in sex-specific FDLE cells

**Figure 5:**
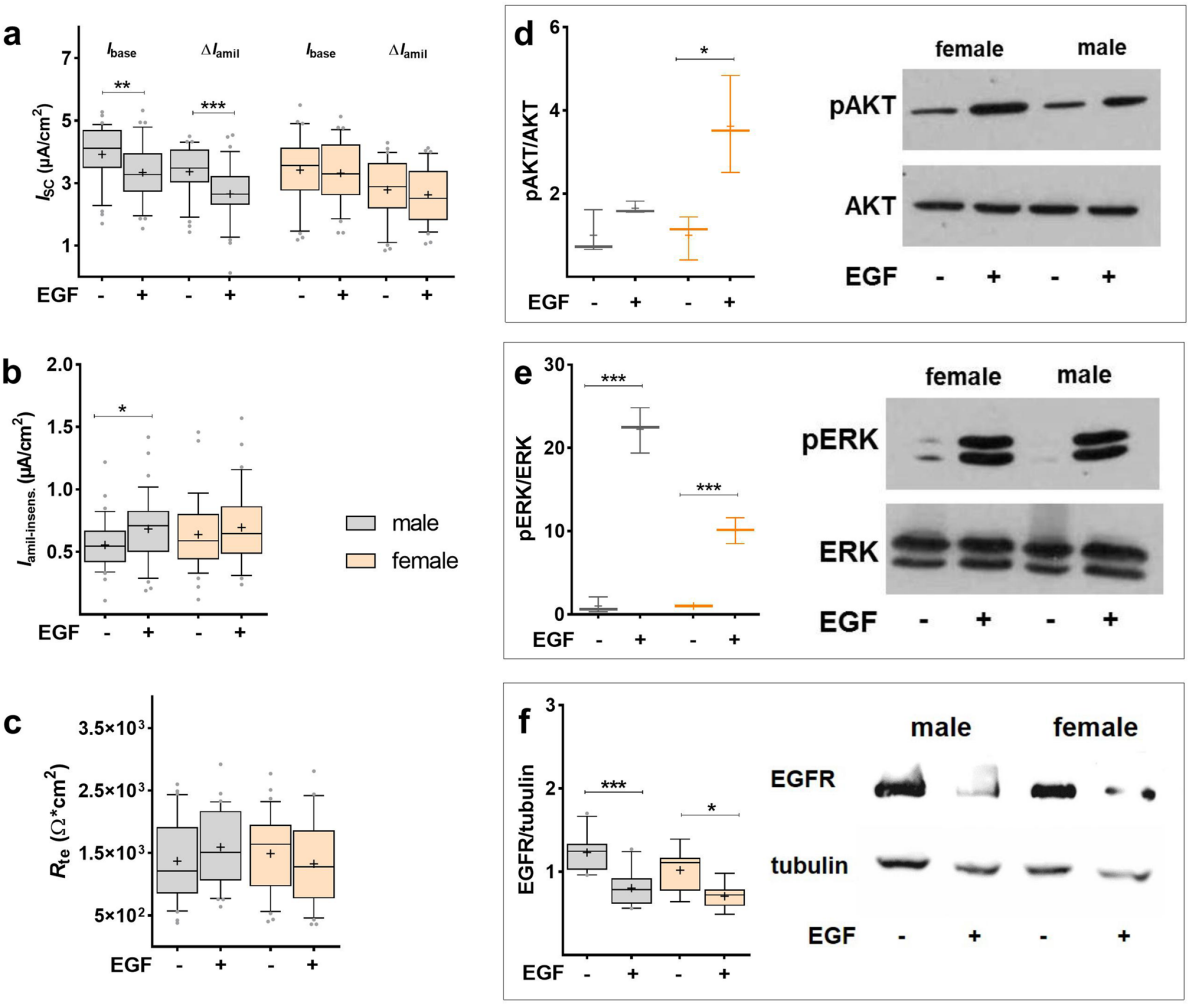
Acute sex-specific effects of EGF via AKT-ERK1/2 signaling. FDLE cells were cultured in SF-Med and stimulated with EGF for 45 min. Data are displayed as box and whiskers with the 10–90 percentile, mean (+) and median (horizontal line). (**a**) In male FDLE cells, EGF significantly decreased *I*_base_ and Δ*I*_amil_ after 45 min (n = 32–37; **p < 0.01; ***p < 0.001 by *t*-test), while female FDLE cells were not significantly affected by acute EGF application. (**b**) EGF acutely increased *I*_amil-insens_ in male FDLE cells (*p < 0.05), while *R*_te_ (**c**) was not affected within the experimental time frame. (**d**) EGF significantly increased AKT phosphorylation within 15 min only in female FDLE cells (n = 3; *p < 0.05 by *t*-test). (**e**) In contrast, EGF significantly increased ERK1/2 phosphorylation within 15 min in both, male and female FDLE cells (n = 3; ***p < 0.001 by *t*-test). (**f**) Short-term EGF exposure decreased EGFR protein expression (n = 10/9; *p < 0.05; ***p < 0.001 by *t*-test). (Grey boxes) male; (yellow boxes) female.

Previous studies suggested a biphasic response with acute EGF effects differing from chronic effects. Sex-specific FDLE cells were thus cultured in SF-Med for 24 h prior to the addition of EGF for 45 min. In accordance to the chronic EGF treatment, *I*_base_ and Δ*I*_amil_ were significantly decreased by EGF after 45 min (*p* < 0.01; *p* < 0.001; Fig. 5a). However, this effect was only observed in male FDLE cells, while female FDLE cells were not affected by acute EGF addition. Moreover, *I*_amil-insens_ was significantly increased by EGF only in male cells (*p* < 0.05; Fig. 5b). Barrier function was not affected by short-term EGF treatment since the *R*_te_ was not different compared to controls or between sexes (Fig. 5c). Western blot demonstrated a significantly higher AKT phosphorylation in female FDLE cells after acute EGF treatment that was not observed in males (*p* < 0.05; Fig. 5d). ERK phosphorylation was significantly elevated by acute EGF application in both male and female cells, although the response to EGF with regard to ERK1/2 phosphorylation was stronger in male cells (*p* < 0.001; Fig. 5e). Since AKT phosphorylation is suggested to increase ENaC activity, and ERK1/2 phosphorylation downregulates ENaC, elevated pAKT in combination to a lower increase of pERK1/2 in females possibly prevents the decrease in ENaC activity that is observed in males after acute EGF treatment. These results show that at least for the acute effects of EGF sex-specific differences were observed with regard to Na^+^ and Cl^−^ transport. EGFR protein expression was also decreased by short-term EGF exposure by approximately 30% in both male and female FDLE cells (*p* < 0.05; *p* < 0.001; Fig. 5f).

**Figure 6:**
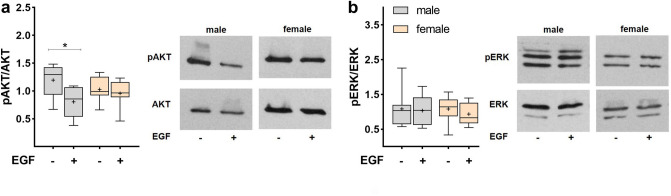
Effect of chronic EGF exposure on AKT-ERK1/2 phosphorylation. Data are displayed as box and whiskers with the 10–90 percentile, mean (+) and median (horizontal line). FDLE cells were cultured in SF-Med and stimulated with EGF for 24 h. (**a**) EGF reduced AKT phosphorylation in male FDLE cells (n = 8; *p < 0.05 by *t*-test). (**b**) In contrast, chronic EGF exposure had no significant effect on ERK1/2 phosphorylation (n = 8).

In contrast to the acute effects of EGF, AKT phosphorylation was reduced in male FDLE cells after chronic (24 h) EGF treatment (*p* < 0.05; Fig. 6a), while AKT phosphorylation in females was not different between EGF-treated and untreated cells. Furthermore, ERK phosphorylation was not different in chronically EGF-treated and controls in both male and female FDLE cells (Fig. 6b). Thus, the acute effects of EGF on AKT/ERK phosphorylation differ from its chronic effects.

### EGF affects barrier permeability and metabolic activity in sex-specific FDLE cells

**Figure 7:**
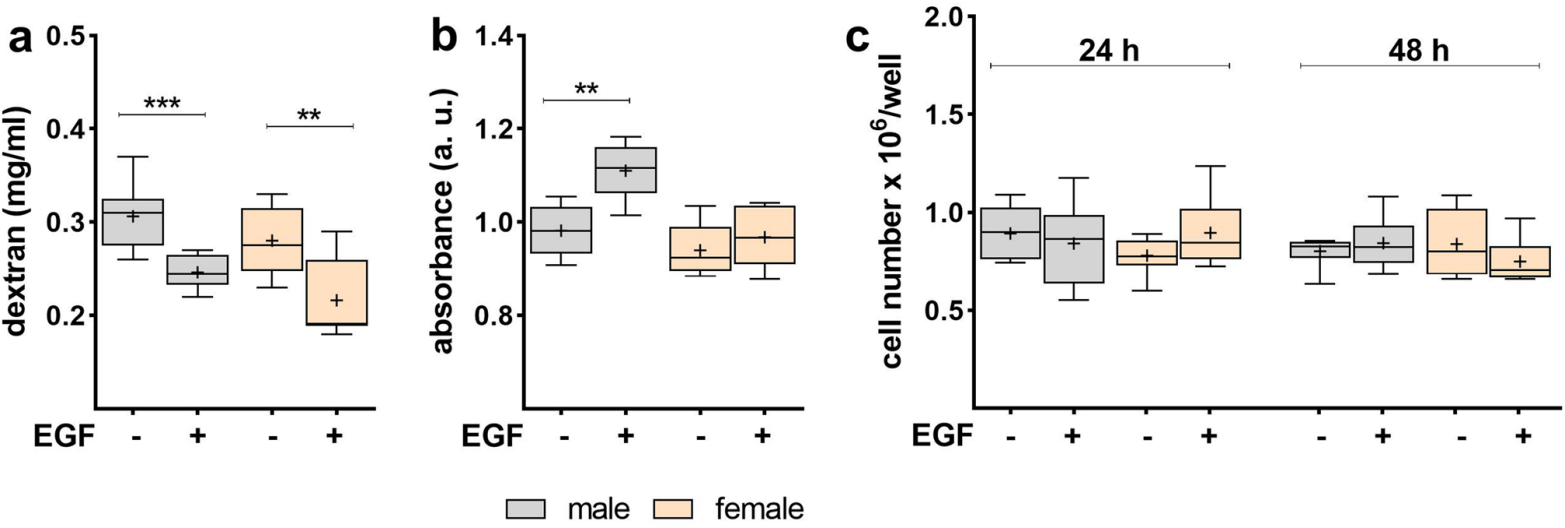
EGF decreased permeability in both sexes, but increased metabolic activity only in males. FDLE cells were cultured in SF-Med and stimulated with EGF for 24 h. Data are displayed as box and whiskers with the 10–90 percentile, mean (+) and median (horizontal line). (**a**) FITC-dextran assay demonstrated a significantly reduced permeability induced by EGF in male and female FDLE cells (n = 8; **p < 0.01; ***p < 0.001 by *t*-test). (**b**) EGF further increased the metabolic activity in male FDLE cells, in contrast to female cells, as shown with a MTT assay (n = 6; **p < 0.01 by *t*-test). (**c**) The cell number was not altered by EGF addition in male and female FDLE cells after 24 and 48 h (n = 6–8). (Grey boxes) male; (yellow boxes) female.

As shown above, chronic EGF treatment strongly elevated *R*_te_ suggesting an improved barrier permeability. To confirm this observation a FITC-dextran assay was conducted showing a significantly lower epithelial permeability induced by EGF in both male and female FDLE cells (*p* < 0.001; Fig. 7a). The elevated *R*_te_ and the reduced permeability support an improved barrier function induced by EGF. On the other hand, metabolic activity determined by an MTT assay was elevated only in male cells, while female cells were not affected by EGF (*p* < 0.01; Fig. 7b). FDLE cell numbers after 24 and 48 h of EGF treatment were unchanged in both male and female cells, suggesting that EGF did not stimulate proliferation of epithelial monolayers (Fig. 7c).

### Response to EGF in epithelial-mesenchymal co-cultures

**Figure 8:**
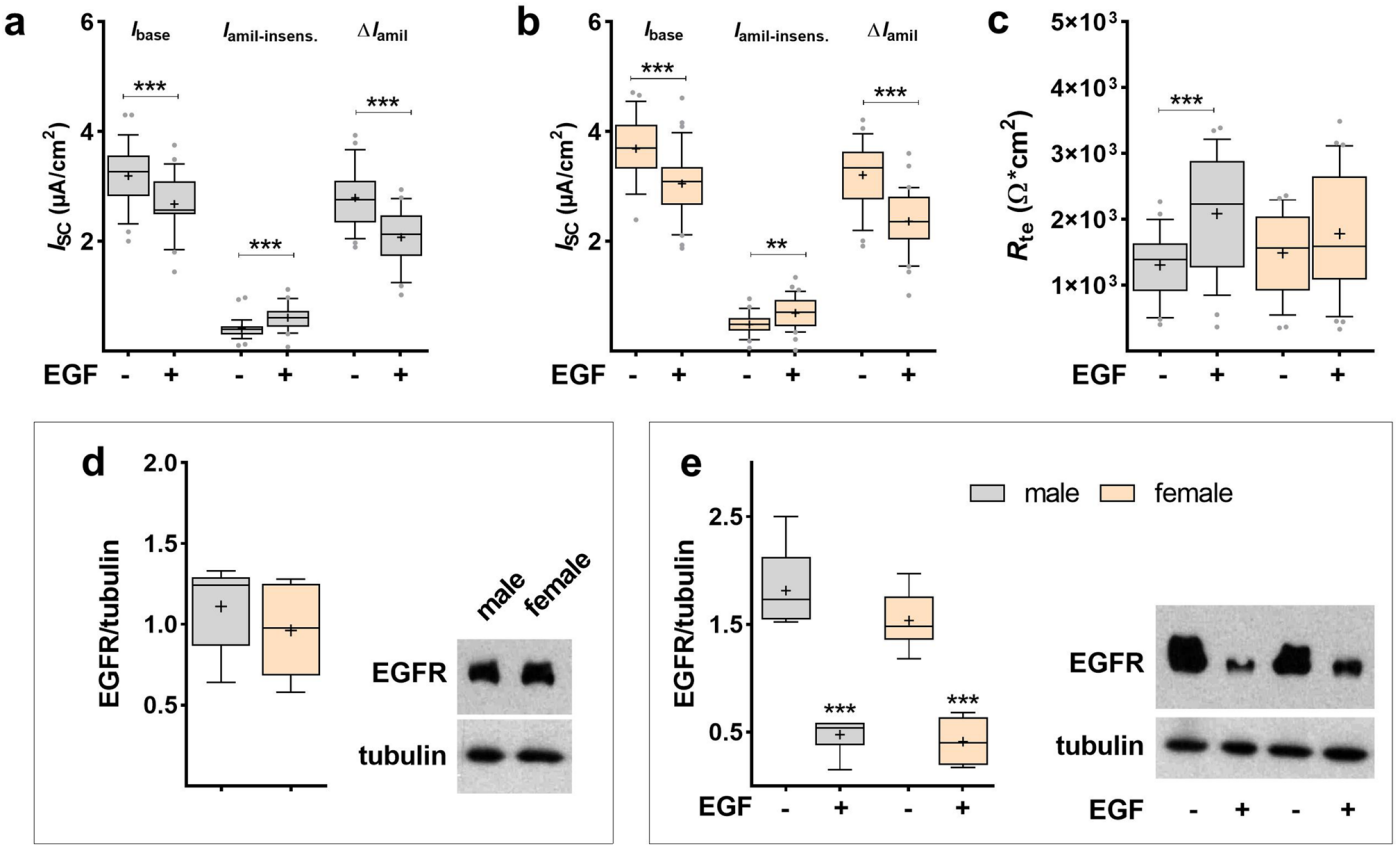
EGF decreased Na^+^ transport in epithelial-fibroblast co-cultures of both sexes. FDLE cells were co-cultured with fetal lung fibroblasts in SF-Med and stimulated with EGF for 24 h. Data are displayed as box and whiskers with the 10–90 percentile, mean (+) and median (horizontal line). In male (**a**) and female (**b**) FDLE cells, EGF significantly decreased *I*_base_ and Δ*I*_amil_ and increased *I*_amil-insens_ (n = 26–32; **p < 0.01; ***p < 0.001 by *t*-test). (**c**) EGF significantly increased *R*_te_ only in male cells (***p < 0.001). (**d**) EGFR protein expression was not different between male and female fetal lung fibroblasts cultured in serum-supplemented medium (n = 6). (**e**) EGF application to fetal lung fibroblasts cultured in SF-Med strongly decreased EGFR protein levels after 24 h in both, male and female fibroblasts (n = 4; ***p < 0.001 by *t*-test). (Grey boxes) male; (yellow boxes) female.

EGF is known to stimulate the epithelial-mesenchymal interaction during fetal lung development. We therefore treated co-cultures of sex-specific FDLE cells and sex-specific fetal lung fibroblasts with EGF for 24 h. EGF significantly reduced *I*_base_ and Δ*I*_amil_ in both sexes (*p* < 0.001; Fig. 8a, b). Moreover, *I*_amil-insens_ was increased by EGF in male and female FDLE cells as shown above, lacking sex-specific effects (*p* < 0.01; *p* < 0.001; Fig. 8a, b). Epithelial barrier function with regard to *R*_te_ was highly increased by EGF only in males (*p* < 0.001; Fig. 8c), although the same trend was observed in females. EGFR protein expression in sex-specific fetal lung fibroblasts cultured in the presence (Fig. 8d) or absence of fetal serum (Fig. 8e) was not different between male and female fibroblasts. EGF application again significantly reduced EGFR protein levels in both male and female fibroblasts (*p* < 0.01; Fig. 8e).

### Effects of EGF after inhibition of EGFR with Erlotinib

**Figure 9:**
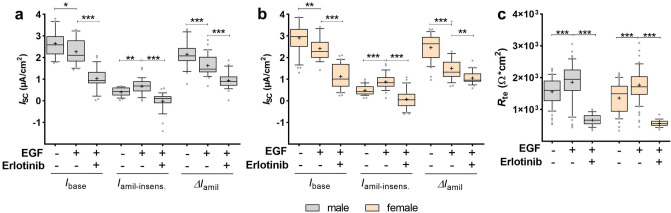
EGFR inhibitor prevented the effect of EGF on *I*_amil-insens_ and *R*_te_, but not on Na^+^ transport. FDLE cells were cultured in SF-Med and stimulated with EGF with or without erlotinib for 24 h. Data are displayed as box and whiskers with the 10–90 percentile, mean (+) and median (horizontal line). EGF significantly decreased *I*_base_ and Δ*I*_amil_ in both, male (**a**) and female (**b**) FDLE cells, which was further decreased by erlotinib (n = 26–31; *p < 0.05; **p < 0.01; ***p < 0.001 by *t*-test). In contrast, erlotinib prevented the significantly increased *I*_amil-insens_ induced by EGF in both sexes. (**c**) Erlotinib further prevented the significant increase of *R*_te_ induced by EGF in both sexes (***p < 0.001). (Grey boxes) male; (yellow boxes) female.

Inhibition of EGFR with Erlotinib did not prevent the decrease of Na^+^ transport induced by EGF, but even further decreased *I*_base_ and Δ*I*_amil_ in both sexes (*p* < 0.05; *p* < 0.01; *p* < 0.001; Fig. 9a, b). On the other hand, Erlotinib prevented the increase of *I*_amil-insens_ induced by EGF (*p* < 0.01; *p* < 0.001; Fig. 9a, b). Furthermore, the significant increase of *R*_te_ induced by EGF was completely abolished by inhibition with Erlotinib (*p* < 0.001; Fig. 9c). Thus, Erlotinib prevents only some of the EGF-induced effects on epithelial ion transport, whereas other effects were even amplified.

### Effects of EGF in combination with female sex steroids

**Figure 10:**
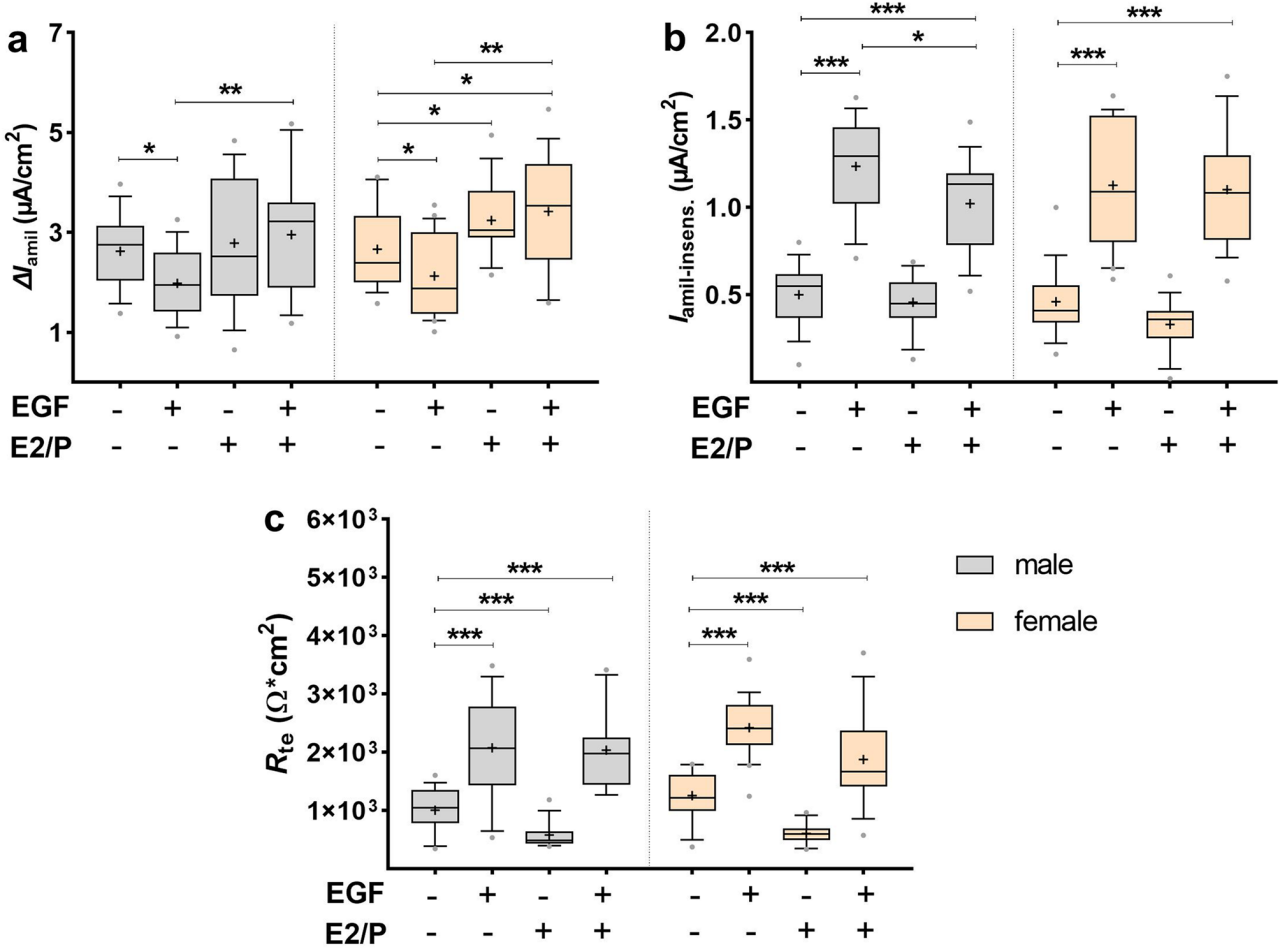
Female sex steroids prevented the reducing effect of EGF on Na^+^ transport. FDLE cells were cultured in SF-Med and stimulated with EGF with or without female sex steroids (E2/P). Data are displayed as box and whiskers with the 10–90 percentile, mean (+) and median (horizontal line). (**a**) EGF significantly decreased Δ*I*_amil_ in male FDLE cells, which was reversed by the addition of E2/P (n = 16–18; *p < 0.05; **p < 0.01 by *t*-test). Moreover E2/P stimulated Δ*I*_amil_ in females and prevented the reduction induced by EGF (n = 15–18; *p < 0.05; **p < 0.01). (**b**) EGF significantly increased *I*_amil-insens_ in both male and female FDLE cells, which persisted in the presence of E2/P (*p < 0.05; ***p < 0.001). (**c**) EGF significantly increased *R*_te_, while E2/P reduced *R*_te_ in male and female FDLE cells. In addition, EGF prevented the decrease of *R*_te_ induced by E2/P (**p < 0.01; ***p < 0.001). (Grey boxes) male; (yellow boxes) female.

Sex steroid, especially female sex steroids, have been previously shown by us to stimulate Na^+^ transport in FDLE cells^15,29^. Whether this stimulation is affected by the presence of EGF was analyzed in male and female FDLE cells treated with E2/P and EGF, alone or in combination. Female sex steroids prevented the reducing effect of EGF on Na^+^ transport. EGF significantly decreased Δ*I*_amil_ in male FDLE cells, which was reversed by the addition of E2/P (*p* < 0.05; *p* < 0.01; Fig. 10a). Moreover E2/P stimulated Δ*I*_amil_ in females and prevented the reduction induced by EGF (*p* < 0.05; *p* < 0.01). EGF significantly increased *I*_amil-insens_ in both male and female FDLE cells, which persisted in the presence of E2/P (*p* < 0.05; *p* < 0.001; Fig. 10b). EGF significantly increased *R*_te_ as shown above, while E2/P reduced *R*_te_ in male and female FDLE cells. EGF prevented this decrease of *R*_te_ induced by E2/P in both male and female FDLE cells (*p* < 0.01; *p* < 0.001; Fig. 10c). In conclusion, the presence of female sex steroids alters the response to EGF, independent of the cells’ sex. Notably, the concentration of female sex steroids in utero is equal for both sexes, as they are produced by the maternal placenta. Therefore, a sex specific effect of EGF in combination with female sex steroids is unlikely, at least for the fetal situation.

### Response of sex-specific FDLE cells to other ErbB ligands

**Figure 11:**
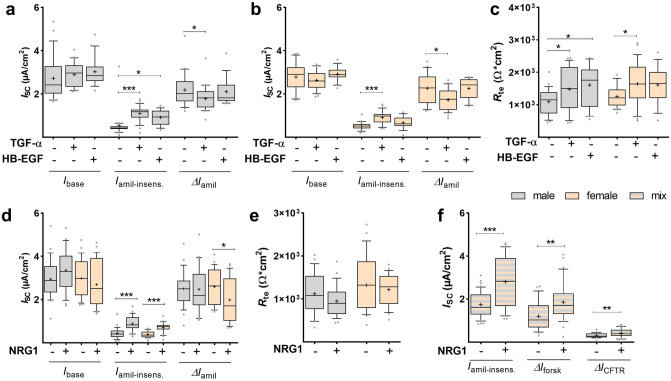
ErbB ligands equally increased amiloride-insensitive *I*_SC_ in both sexes. FDLE cells were cultured in SF-Med and stimulated with TGF-α, HB-EGF or NRG1 for 24 h. Data are displayed as box and whiskers with the 10–90 percentile, mean (+) and median (horizontal line). TGF-α increased *I*_amil-insens_ and decreased Δ*I*_amil_ in male (**a**) and female (**b**) FDLE cells (n = 23–38; ***p < 0.001 by *t*-test). HB-EGF increased *I*_amil-insens_ in male cells (n = 15–23; *p < 0.05). (**c**) TGF-α increased *R*_te_ in male and female cells, and HB-EGF increased *R*_te_ in male cells, with the same trend observed in females (*p < 0.05). (**d**) NRG1 increased *I*_amil-insens_ in male and female cells, while NRG1 decreased Δ*I*_amil_ in female FDLE cells (n = 25–27; *p < 0.05; ***p < 0.001). (**e**) *R*_te_ was not affected by NRG1 in male and female cells. (**f**) NRG1 increased *I*_amil-insens_, Δ*I*_forsk_ and Δ*I*_CFTR_ in mixed FDLE cells (n = 39–43; **p < 0.01; ***p < 0.001). (Grey boxes) male; (yellow boxes) female; (white square) mix.

Finally, other ErbB receptor ligands were analyzed in sex-specific FDLE cells. TGF-α exerted no effect on *I*_base_ in male and female cells (Fig. 11a, b), which is in contrast to EGF as detailed above. However, Δ*I*_amil_ was significantly decreased and *I*_amil-insens_ strongly increased by TGF-α in both sexes (*p* < 0.05; *p* < 0.001; Fig. 11a, b). We assume that the increased *I*_amil-insens_ together with the small decrease of Δ*I*_amil_ counterbalanced total *I*_SC_ and thus resulted in no net difference of *I*_base_. In accordance to EGF, TGF-α significantly increased *R*_te_ (*p* < 0.05; Fig. 11c). Thus, both EGFR (ErbB1) ligands, EGF and TGF-α, equally affect Na^+^ and Cl^−^ transport, and barrier function in a sex-independent manner, even though not to the same degree. HB-EGF increased *I*_amil-insens_ and *R*_te_ in males, with a similar trend in females (*p* < 0.05; Fig. 11a, c), while *I*_base_ and Δ*I*_amil_ were not affected, neither in male nor in female FDLE cells (Fig. 11a–c). ErbB2 ligand NRG1 further increased *I*_amil-insens_ in male and female cells, while Δ*I*_amil_ was reduced only in females (*p* < 0.05; *p* < 0.001; Fig. 11d). Barrier function with regard to *R*_te_ was not affected by NRG1 (Fig. 11e). In mixed FDLE cells, NRG1 further significantly increased Δ*I*_forsk_ and Δ*I*_CFTR_ (*p* < 0.01; Fig. 11f). NRG1 therefore shares the Cl^−^ transport stimulating properties of EGF, which were sex-independent.

## Discussion

We aimed to determine whether EGF, a prominent mitogen during fetal lung development, exerts sex-specific effects on epithelial Na^+^ transport in fetal alveolar cells. EGF strongly reduced the ENaC subunit mRNA levels in both male and female FDLE cells. This was corroborated by a markedly reduced Na^+^ transport and ENaC activity, while amiloride-insensitive pathways as well as the barrier function (*R*_te_) were raised by EGF. Notably, CFTR-mediated Cl^−^ transport was elevated only in female cells, while CFTR mRNA levels were increased in male and female cells. The effects of EGF were reproduced with other ligands of the EGFR (TGF-α), ErbB3 (NRG1) and ErbB4 (HB-EGF, NRG1), diminishing Na^+^ transport in FDLE cells. In contrast to chronic effects of EGF after 24 h, acute effects of EGF were sex-specific, because Na^+^ transport was reduced only in males. Furthermore, pAKT was elevated only in female cells in response to acute EGF addition, while pERK1/2 was increased in both male and female cells. The study thus showed certain sex- and time-dependent effects on epithelial ion transport induced by EGF, while other analyzed aspects were similar between male and female FDLE cells.

Prior studies reported contradictive results about the effect of EGF on epithelial Na^+^ transport. In alveolar epithelial cells derived from adult male rats EGF was shown to increase net Na^+^ flux and the activity of the Na,K-ATPase via basolaterally located EGFR^32^. The elevated Na^+^ transport was attributed to an increased *Na,K-ATPase-α*_1_ and *-β*_1_ mRNA and protein expression^33^. Furthermore, in adult rat ATII cells EGF treatment increased the whole-cell conductance and density of ENaC channels, whereas mRNA expression of all three ENaC subunits was decreased^34^. In contrast, the majority of studies observed a negative effect of EGF on Na^+^ transport. EGF markedly and quickly decreased ENaC activity by lowering its open probability, paralleled by a reduction of plasma membrane phosphatidylinositol 4,5-bisphosphate (PIP_2_) levels in ENaC-reconstituted CHO cells^35^. Anionic phospholipids such as PIP_2_ and phosphatidylinositol 3,4,5-trisphosphate (PIP_3_) were shown to regulate ENaC and the stimulation of PLC by EGFR activation decreased PIP_2_ and consequently ENaC activity^35–37^. EGF rapidly diminished amiloride-sensitive Na^+^ currents in perfused rabbit and mouse collecting tubules^38,39^, which was attributed to a rapid phosphorylation of ERK1/2. EGF further raised basal intracellular Ca^2+^ levels after 24 h and reduced ENaC function by a Ca^2+^-mediated process that affected trafficking and surface expression of ENaC in a human nasal airway epithelial cell line derived from a CF patient^25^. These contrasting results may suggest cell and/or tissue specific EGF-dependent regulation of ENaC activity. It is proposed that EGF increases Na^+^ absorption in the airways and the intestine, whereas it supposedly reduces Na^+^ transport in CCD cells, CF cells, CHO cells overexpressing ENaC and EGFR, and other kidney-derived cells^40^. In contrast to this, we observed an antagonizing effect of EGF on Na^+^ transport in alveolar epithelial cells of fetal origin, which suggests that EGF effects also depend on the developmental stage of the cells. Furthermore, chronic incubation with EGF reduced Na^+^ transport in both male and female cells, while short-term incubation reduced ENaC activity only in males. To our knowledge, this is the first study demonstrating a sex-specific effect of EGF on Na^+^ transport. Different modes of EGF-dependent modulation of ENaC-mediated Na^+^ absorption have been proposed. EGF caused a transient increase in Na^+^ transport in A6 cells, which was PI3-K dependent, followed by a chronic reduction of ENaC membrane abundance mediated through MAPK1/2^40,41^. Inhibition of MEK1/2, thereby preventing ERK1/2 phosphorylation, stimulated the acute increase and abolished the chronic decrease of Na^+^ transport in response to EGF^40^. An increased pERK1/2 was demonstrated in male and female cells after short-term EGF exposure, whereas pAKT was significantly increased only in females. While activation of PI3-K/AKT signaling has been associated with enhanced ENaC activity, ERK1/2 signaling usually results in a decreased ENaC function. We suggest that the acutely increased pAKT counterbalances the diminishing effect of induced ERK1/2 signaling, resulting in no net changes of Na^+^ transport in females, whereas this compensatory effect is lacking in males leading to a decreased Na^+^ transport. Unlike the acute effects, chronic EGF application resulted in a reduction of pAKT in males, while no difference was observed for pERK1/2 in male and female FDLE cells after 24 h.

Importantly, EGF strongly stimulated the amiloride-insensitive fraction of epithelial ion transport. It has been previously shown that FDLE cells exhibit different amiloride-insensitive pathways, including secretion of Cl^−^ and HCO_3_^−^^28,42^. Therein the presence of cAMP-activated Cl^−^ and HCO_3_^−^ secretion across rat FDLE cells was demonstrated, which was mediated via CFTR^42^. Raising intracellular cAMP levels by forskolin after inhibition of ENaC activity with amiloride increased *I*_SC_ in female FDLE cells. Furthermore, CFTR activity was strongly increased by EGF, but only in females. Therefore, EGF increased amiloride-insensitive pathways in both sexes, but this increase was attributable to an enhanced CFTR activity only in female FDLE cells. In contrast to this, CFTR mRNA expression was elevated by EGF in both male and female FDLE cells. Short-term exposure to EGF elevated amiloride-insensitive currents only in males. The effects of EGF on Na^+^ transport and barrier function were normalized by removing EGF from the culture medium for 24 h, while the effect on amiloride-insensitive pathways persisted. In conclusion, EGF affects Na^+^ transport and amiloride-insensitive pathways in both sexes, but with varying magnitude and time specifics. Only few studies dealt with a relationship between EGF and epithelial Cl^−^ secretion. In T84 cells acute EGF chronically increased *I*_SC_ responses to a broad range of secretagogues, and bumetanide, an inhibitor of the Na,K,2Cl-cotransporter, abolished the effect of EGF, suggesting an enhanced Cl^−^ secretion^43^. In addition, Cl^−^ secretion was enhanced by EGF through upregulation of the Ca^2+^-dependent Cl^−^ channel, transmembrane protein 16A (TMEM16A), in the apical membrane of colonic cells^44^. Another group demonstrated that EGF inhibited Ca^2+^-dependent Cl^−^ secretion via stimulation of the PI3-K through recruitment of the regulatory PI3-K subunit p85 to ErbB2 in colonic epithelial cells^45^. Thus, regulation of epithelial Na^+^ transport and Cl^−^ secretion by EGF is not consistent between different studies and cell-, sex- and developmental-specific effects have to be considered.

Notably, prolonged EGF application strongly stimulated *R*_te_ in both male and female cells, suggesting an improved barrier integrity that was confirmed by a permeability assay. Neither metabolic activity nor cell number was affected by EGF, suggesting that the enhanced barrier function is not due to an increased cellular proliferation. Other studies demonstrated that EGF increased *R*_te_ across adult alveolar epithelial cell monolayers and reduced the tight junction permeability^32^, possibly by regulating the expression of certain claudins^46^. In addition, EGF has been shown to be an effective intestinal regulator helping to protect intestinal barrier integrity via binding to EGFR and subsequent activation of MAPK/ERK, PI3-K/AKT, PLC-γ/PKC (protein kinase C), and STATS (signal transducers and activators of transcription substrates) signal pathways^47^.

In general, EGFR signaling is associated with lung growth and maturation, and a reduced EGFR expression was observed in hypoplastic fetal lungs^48,49^. In addition, lower EGF levels in the bronchoalveolar lavage fluid at birth were detected in infants with RDS or bronchopulmonary dysplasia compared with control infants^50^. On the other side, EGFR overactivation has been associated with acute respiratory distress syndrome (ARDS) and fibrosis development^51,52^. Protein expression of EGFR was higher in female FDLE cells. It has been shown previously that estrogen increases the uterine levels of EGFR^53^ and that EGFR density is higher in the female fetal rabbit lung, possibly due to a negative impact of androgens^54^. Chronic androgen treatment has been shown to reduce EGFR activity inhibiting surfactant protein gene expression in ATII cells and fetal lungs^55–57,57^. Serum-free cultivation of FDLE cells abolished the sex difference in EGFR protein expression. Thus, sex differences in EGFR protein expression were affected by the presence of fetal serum in the culture and possibly the fetal circulation. Both, the mRNA and protein expression of EGFR was strongly decreased by EGF in both sexes. This was observed after acute (45 min) and chronic (24 h) EGF treatment, although the extent was more pronounced after prolonged application. Similarly, EGFR protein expression was reduced by EGF in fetal lung fibroblasts, while no sex-specific expression was observed in the latter cell type. It can be assumed that this negative feedback regulation prevents hyperproliferation as shown in preterm baboon lungs with hyaline membrane disease possibly induced by elevated levels of the EGFR ligand TGF-α^58^.

Studies showed that EGF increased thymidine incorporation and DNA accumulation in fetal lung fibroblasts and ATII cells, and elevated choline incorporation into surfactant phosphatidylcholine^23^. EGF positively influenced the epithelial-mesenchymal interaction, and EGFR binding in fetal rat lung fibroblasts peaks on embryonic day 18–19, prior to surfactant surge^56^. EGFR inactivation or EGFR deficiency resulted in ATII cell immaturity apparent from an increased glycogen content and a reduced number of lamellar bodies^59^. EGF further stimulated *Sftpc* expression in normal but not in EGFR-deficient lungs^60^. It is suggested that EGF may elaborate a fibroblast-derived factor that stimulates surfactant synthesis in ATII cells^61^. We also demonstrated an enhanced mRNA expression of the surfactant proteins in response to EGF that was observed in male and female FDLE cells, lacking any pronounced sex difference. Previous studies observed sex differences in surfactant synthesis with males lagging behind, although both sexes achieved comparable responses in late gestation^24^. Our results could thus be explicable by the fact that FDLE cells are isolated from late gestation lungs during the saccular stage, while the surge in surfactant synthesis occurs during the canalicular stage. Furthermore, the prior studies addressed surfactant synthesis and not surfactant protein mRNA expression possibly explaining the differential outcomes.

As noted above, EGF supposedly exerts its effect on lung epithelial cells by affecting the epithelial-mesenchymal relationship. EGF has been shown to increase the proliferation of epithelial and mesenchymal cells in monoculture, and within the embryonic lung, the mesenchyme represents the major source of EGF as a paracrine factor acting on epithelial cells expressing EGFR^17^. Thereby epithelial proliferation and differentiation is stimulated. Due to this, we co-cultured FDLE cells with fetal lung fibroblasts in a sex-specific manner and repeated the preceding experiments. Despite the presence of fibroblasts, the effects of EGF on Na^+^ transport were essentially the same as we observed before. The presence of fibroblasts did not affect the response of epithelial Na^+^ transport to EGF, ruling out any indirect stimulating effects in this context.

Inhibition of EGFR with erlotinib prevented only some effects of EGF. Erlotinib inhibited the increase of amiloride-insensitive pathways and barrier function, while the reduction of Na^+^ transport was not affected. It is unclear why erlotinib is unable to prevent the reduced Na^+^ transport. However, the effects of erlotinib were not sex-specific. Erlotinib is a reversible inhibitor of EGFR that also exerts inhibitory activity on purified ErbB2^62^. The inhibitory effect of erlotinib in ErbB2-expressing cells is suggested to arise at least in part through direct interaction with ErbB2 rather than indirectly through a process, that requires the presence of EGFR. Since ligandless ErbB2 also dimerizes with ErbB3 or ErbB4 and thereby exerts diverging biological actions, it might be conceivable that some EGF effects were affected by erlotinib while others differed.

Finally, female sex steroids prevented the reducing effect of EGF on Na^+^ transport in both male and female FDLE cells, while the effects of EGF on barrier function and Cl^−^ transport persisted in the presence of female sex steroids. Since the fetal concentration of female sex steroids is equal for both sexes, as they are provided by the maternal placenta, no sex-specific effect of EGF in combination with female sex steroids is assumed. Concerning male sex steroids, no androgen receptor-specific mRNA expression was detected in male or female FDLE cells^15^. Thus, FDLE do not express androgen receptors during the saccular stage at which cell isolation was performed. Furthermore, flutamide, an androgen receptor antagonist showed no effect on Na^+^ transport in FDLE cells^15^. Together, the inability of flutamide to affect Na^+^ transport and the undetectable androgen receptor expression suggest a negligible impact of androgens on Na^+^ transport in FDLE cells.

## Conclusions

EGF strongly reduced the ENaC mRNA expression and channel activity in both male and female FDLE cells. In contrast, amiloride-insensitive pathways as well as the barrier function were raised by EGF. While chronic effects were not sex-specific, short-term effects were limited to males. In conclusion, we showed certain sex- and time-dependent differences in epithelial ion transport induced by EGF, while other analyzed aspects were similar between male and female FDLE cells. From the results obtained in this study it cannot be assumed that EGF is responsible for the sex specific differences in Na^+^ transport observed in FDLE cells.

## Supplementary Information

Supplementary Figures.

## Data Availability

All data generated or analyzed during this study are either included in this published article or can be obtained from the corresponding author on reasonable request.
