# Supplementary material for: Epidermal growth factor strongly affects epithelial Na+ transport and barrier function in fetal alveolar cells, with minor sex-specific effects
Source: Sci Rep. 2021 Aug 5;11:15951. doi: 10.1038/s41598-021-95410-y (PMC8342687; doi:10.1038/s41598-021-95410-y)
Supplement: Supplementary file 1 — Supplementary Figures. [file 41598_2021_95410_MOESM1_ESM.pdf]

Supplement Figure 4a-b

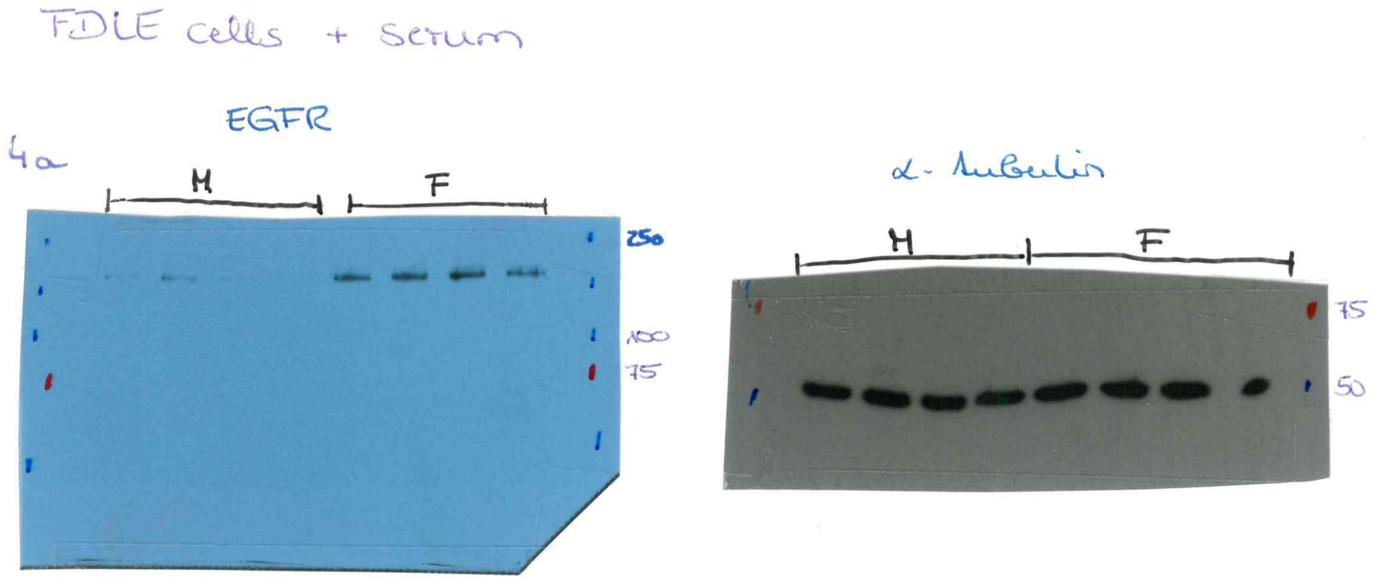

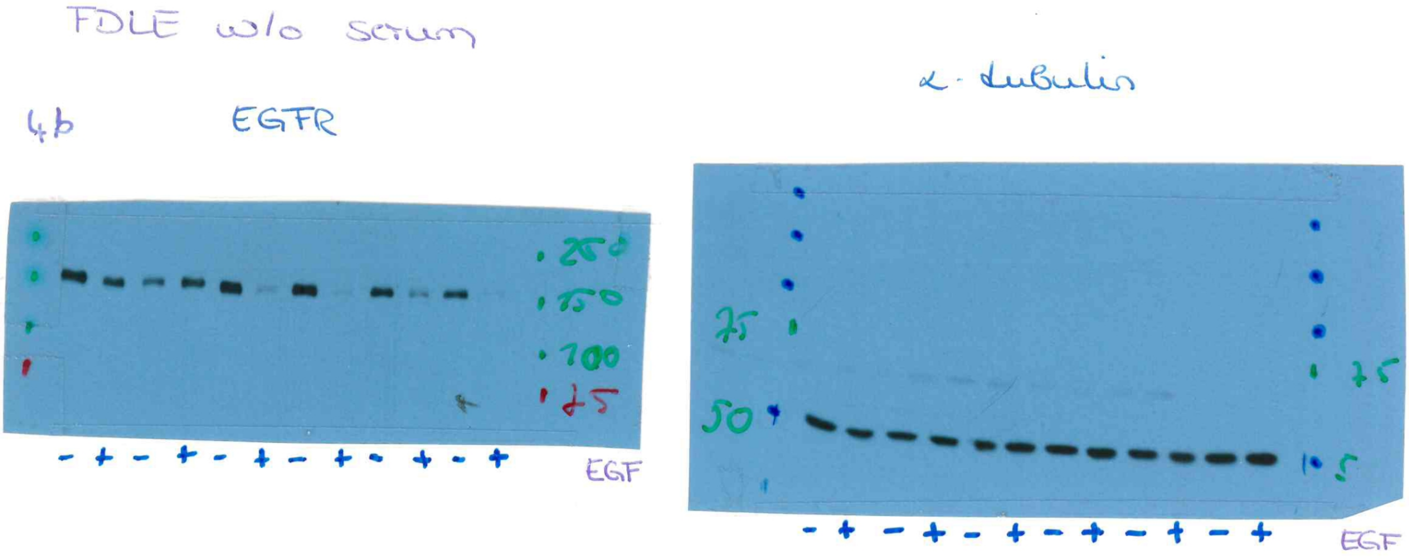

Ladder	CG F1	EGF F1	CG M1	EGF M1	CG F2	EGF F2	CG M2	EGF M2	CG F3	EGF F3	CG M3	EGF M3	Ladder

Supplement Figure 5d-e

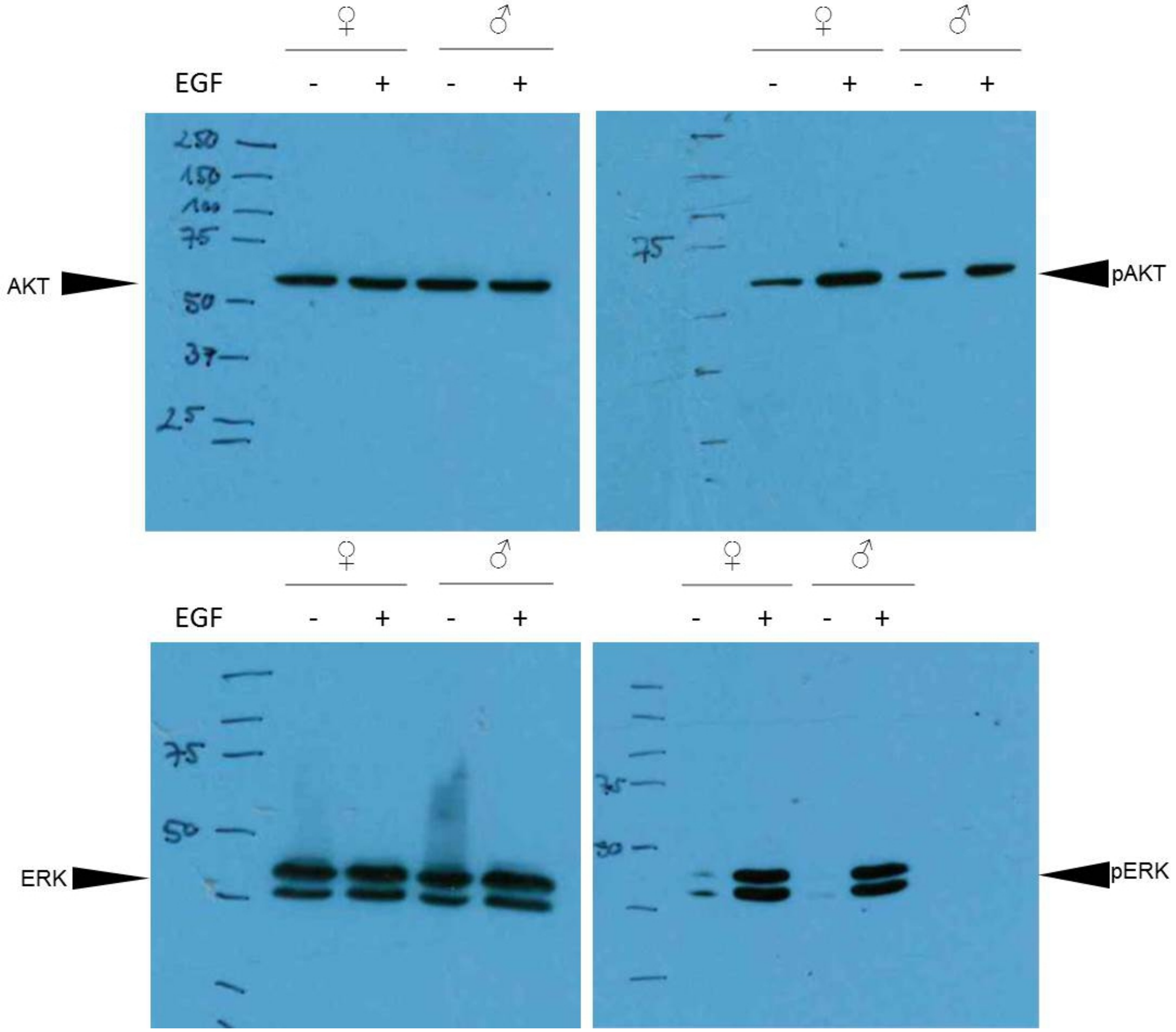

Supplement Figure 5f

Fig. 5f

L	SF-Med M1	EGF M1	SF-Med F1	EGF F1	SF-Med M2	EGF M2	SF-Med F2	EGF F2	L

EGFR (175 kDa)

250
150
100
75
50
37

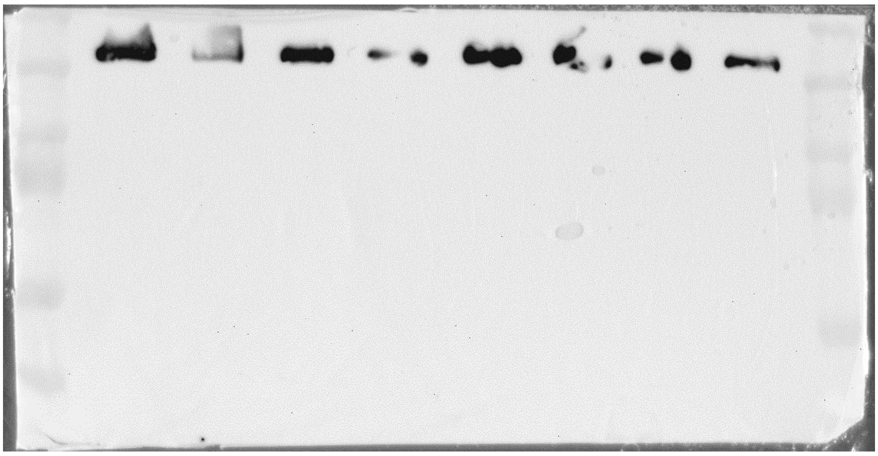

α-tubulin (52 kDa)

250
150
100
75
50
37

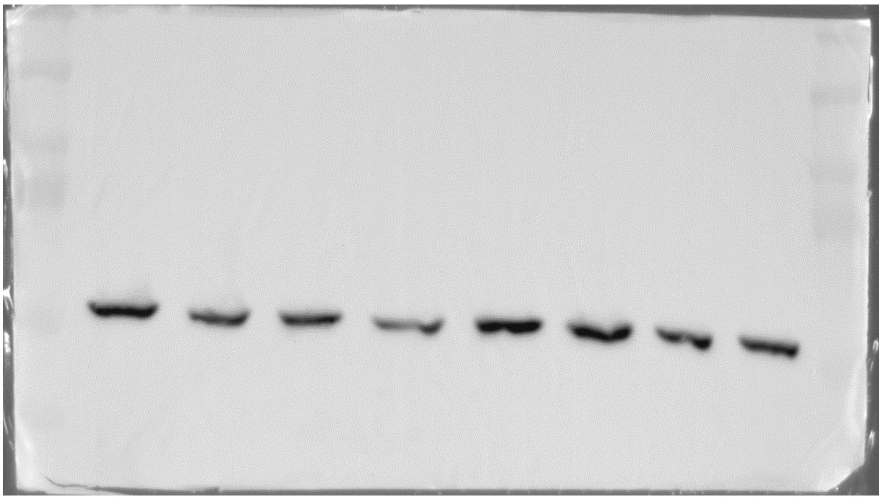

Supplement Figure 6a-b

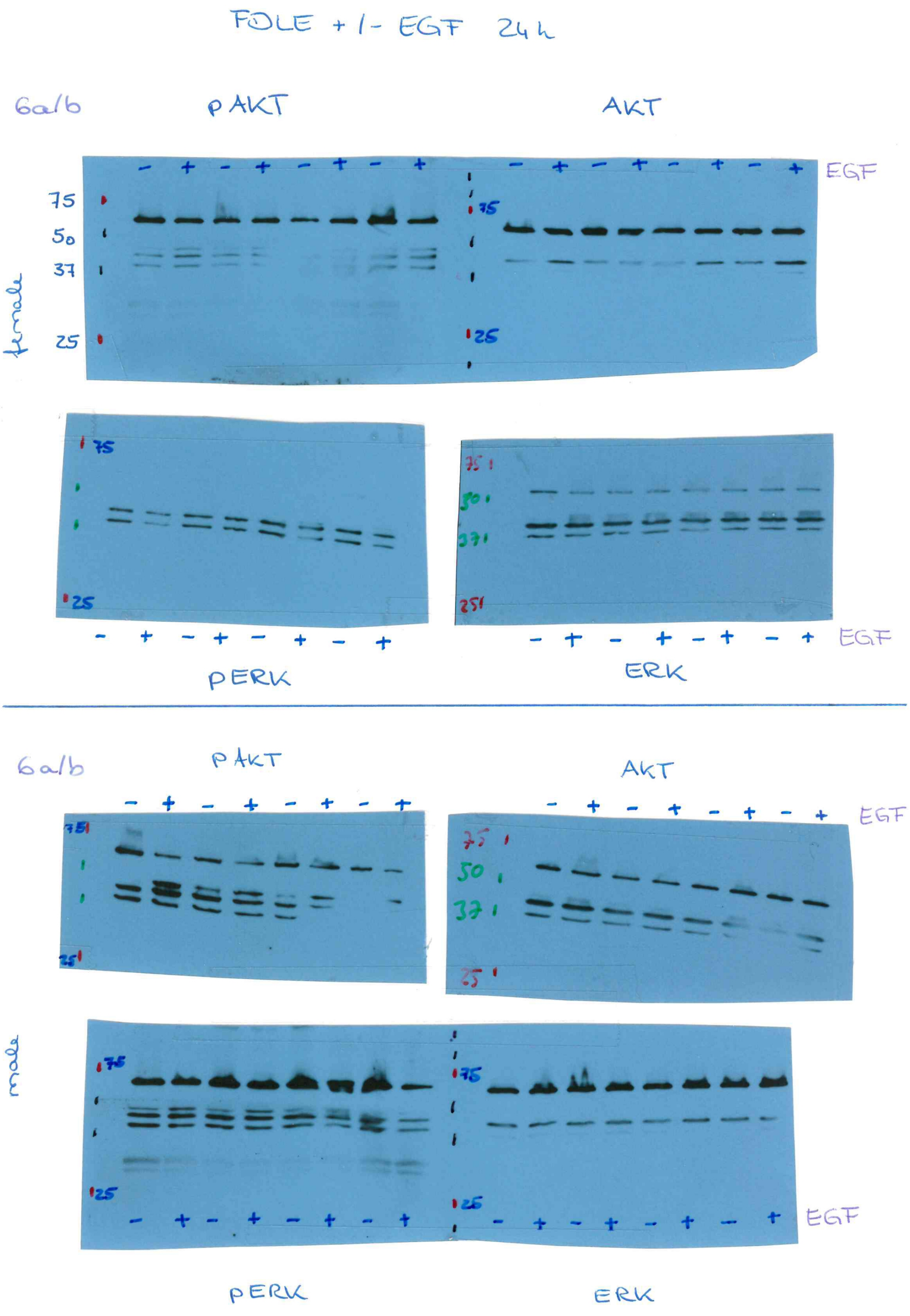

Supplement Figure 8d-e

8 d

fetal lung fibroblasts + serum

1	Ladder	M-ZK1	F-ZK1	M-ZK2	F-ZK2	M-ZK3	F-ZK3	M-ZK4	F-ZK4	M-ZK5	F-ZK5	M-ZK6	F-ZK6

EGFR

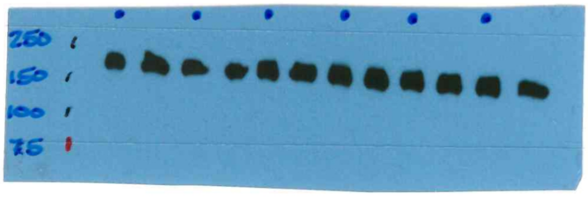

α - tubulin

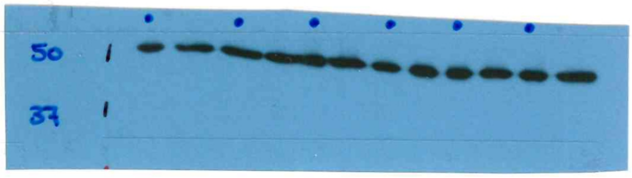

• male

8 e

fetal lung fibroblasts w/o serum

2	Ladder	M-CG 1	M-EGF 1	F-CG 1	F-EGF 1	M-CG 2	M-EGF 2	F-CG 2	F-EGF 2	M-CG 3	M-EGF 3	F-CG 3	F-EGF 3

EGFR

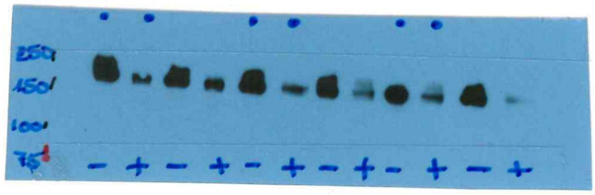

α - tubulin

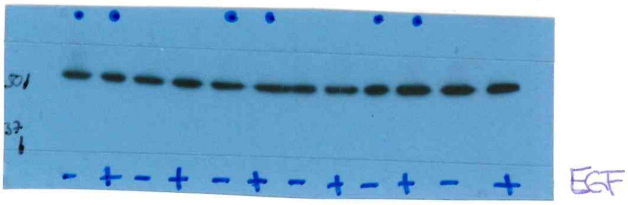

• male
